# Spontaneous correction of scoliosis after curettage of spinal osteoid osteoma: How and when?

**DOI:** 10.1186/s13018-022-03423-8

**Published:** 2022-12-12

**Authors:** Xiyu Pan, Jun Qiao, Zhen Liu, Xu Sun, Zezhang Zhu

**Affiliations:** Division of Spine Surgery, Department of Orthopedic Surgery, Nanjing Drum Tower Hospital, The Affiliated Hospital of Nanjing University Medical School, Zhongshan Road 321, Nanjing, 210008 China

**Keywords:** Scoliosis, Curettage, Osteoid osteoma

## Abstract

**Background:**

Scoliosis behavior after curettage of spinal osteoid osteoma has been not clarified as most studies regarding scoliosis secondary to osteoid osteoma (OO) were case reports. The aims of this study were to investigate (1) clinical and radiographic features of scoliosis secondary to OO; (2) scoliosis behavior after Curettage of OO.

**Methods:**

A retrospective study was performed at patients who were diagnosed as OO clinically or pathologically from July 1998 to December 2019 in a single institution. Age, gender, location of pain, location of lesion and curve pattern of scoliosis were collected preoperatively. Intraoperative blood loss, operation time and surgical complications were documented. VAS scores and curve magnitude were collected pre- and postoperatively and at last follow-up.

**Results:**

The mean operation time was 124 ± 32 min and the average intraoperative blood loss was 274 ± 134 ml. The mean preoperative VAS score was 6.2 ± 2.7, and the mean postoperative VAS score was 2.1 ± 1.8. Thoracic scoliosis was improved from 22.7 ± 10.6° to 6.2 ± 4.3° after operation, and to 4.1 ± 4.3° at last follow-up. Lumbar scoliosis was improved from 18.1 ± 7.4° to 6.7 ± 5.2° after operation, and to 5.3 ± 3.9° at last follow-up. Trunk shift was improved from 34.7 ± 12.4  to 10.5 ± 7.2 mm after operation, and to 8.4 ± 5.6 mm at last follow-up. There was no significant differences as to sagittal radiographic parameters (*P* > 0.05).

**Conclusion:**

Patients with spinal OO had a significantly high incidence of scoliosis. Patients could get rapid relief of pain and scoliosis with low occurrence. Night pain, pain at the concave side of curve, normal sagittal alignment could help differentiate it from scoliosis associated with lumbar disc herniation.

## Introduction

Osteoid osteoma is a primary benign bone lesion, which pathologically features a highly vascularized nidus of connective tissue surrounded by sclerotic bone [1]. The nidus measures about 10 mm in diameter, and the size is the main distinguishing feature between it and osteoblastoma [2]. Predilection sites of the osteoid osteoma are long bones, especially those of the lower extremities [3]. Ten percent of the cases has lesions at spine, and lumbar spine is the most commonly affected [4]. Scoliosis is a common clinical manifestation of OO, with incidence ranging from 20 to 70% [5]. The scoliosis secondary to OO is believed to be pain-evoked reactive. Localized pain is caused by nerve fibers in the nidus. The production of prostaglandin may lead to an increase in vascular pressure, which may produce pain by stimulating afferent nerves around the nidus. Muscle spasm around the nidus then produces scoliosis [6].

In previous studies, as high as 54.5% patients with scoliosis secondary to OO were misdiagnosed, and some were misdiagnosed as adolescent idiopathic scoliosis (AIS), receiving incorrect treatments, such as bracing or acupuncture [7, 8]. Scoliosis would become structural if correct treatment was not implemented in time. Curettage with or without fixation is standard treatment for OO [9, 10]. Theoretically, resection of nidus would eliminate the production of prostaglandin, so that alleviates the muscle spasm. However, scoliosis behavior after curettage has been not clarified as most studies regarding scoliosis secondary to OO were case reports. The aims of this study were to investigate (1) clinical and radiographic features of scoliosis secondary to OO; (2) scoliosis behavior after curettage of OO.

## Methods

### Patients

A retrospective study was performed at patients who were diagnosed as OO clinically or pathologically from July 1998 to December 2019 in a single institution. The inclusion criteria were as follows: (1) with scoliosis larger than 10°; (2) receiving curettage of nidus with or without fixation; (3) more than 2 years follow-up. Patients were excluded when they concomitantly had other types of scoliosis including congenital scoliosis, AIS, disc herniation-related scoliosis and so on.

### Data collection

Age, gender, location of pain, location of lesion and curve pattern of scoliosis were collected preoperatively. Intraoperative blood loss, operation time and surgical complications were documented. VAS scores and curve magnitude were collected pre- and postoperatively and at last follow-up. The study protocol was approved by the Clinical Research Ethics Committee of Nanjing Drum Tower hospital (2021-398-01).

### Radiographic measurements

The following coronal and sagittal parameters were measured at standing posteroanterior and lateral whole spine radiographs preoperative, 3-month-postoperation and at final follow-up:

1. Trunk shift: the deviation of the C7 plumb line from the CSVL;
2. Sagittal vertical axis (SVA): the distance measured between the C7 plumb line (C7PL) and the posterosuperior corner of S1 vertebra. The SVA was positive if the C7PL lied anteriorly to the posterosuperior corner of S1; otherwise, it was negative.
3. Thoracic kyphosis (TK): the angle between the superior endplate of T5 and the inferior endplate of T12;
4. Thoracolumbar kyphosis (TLK): the angle between the superior endplate of T10 and the inferior endplate of L2;
5. Lumbar lordosis (LL): the angle between the superior endplate of L1 and the superior endplate of S1.
6. Thoracic scoliosis: the angle between the superior endplate of most tilted cephalic vertebral and the inferior endplate of most tilted caudal vertebral in the thoracic region.
7. Lumbar scoliosis: the angle between the superior endplate of most tilted cephalic vertebral and the inferior endplate of most tilted caudal vertebral in the lumbar region.

All measurements were performed using Surgimap (version 2.1.2; Spine Software, New York, NY, USA). Two of the authors independently completed the measurements and the mean values were collected for analysis.

### Statistical analysis

SPSS software 17.0 (SPSS Inc., Chicago, IL) was used for statistical analysis. Average values were reported as mean (SD). Summary statistics from the analyses of variance calculations were used to provide 95% confidence intervals for the error in measurements. Anova was used for the comparison of preoperative, postoperative and final follow-up radiographic parameters. The statistical significance was set at *P* < 0.05.

## Results

A total of 32 patients (males, females) were included, with an average age of 18.4 years (range: 11–30 years). 21 patients had OO at lumbar spine while 11 at thoracic spine. 23 patients had double thoracic and lumbar curves and 9 patients had single lumbar curve. All the patients had pain at concave side of the curve. 13 lesions were located at laminar, 5 at facet and 14 at pedicle.

One patient had OO at midline of laminae, and received curettage without fixation. Other 31 patients all underwent curettage with fixation, of which one patient had OOs at T8 and T6 respectively receiving two operations.

The mean operation time was 124 ± 32 min (range: 90–184 min) and the average intraoperative blood loss was 274 ± 134 ml (range: 110–475 ml). The mean follow-up time was 29.4 ± 7.2 months (range: 24–146 months). The mean preoperative VAS score was 6.2 ± 2.7 (range: 3–9), and the mean postoperative VAS score was 2.1 ± 1.8 (range: 0–4).

Thoracic scoliosis was improved from 22.7 ± 10.6° (range: 8–40°) to 6.2 ± 4.3° (range: 0–12°) after operation, and to 4.1 ± 4.3° (range: 0–10°) at last follow-up.

Lumbar scoliosis was improved from 18.1 ± 7.4° (range: 10–31°) to 6.7 ± 5.2° (range: 0–12°) after operation, and to 5.3 ± 3.9° (range: 0–13°) at last follow-up. Trunk shift was improved from 34.7 ± 12.4 mm (range: 10–45 mm) to 10.5 ± 7.2 mm (range: 0–21 mm) after operation, and to 8.4 ± 5.6 mm (range: 0–16 mm) at last follow-up (Table 1). The differences between preoperative and postoperative radiographic parameters as to thoracic scoliosis, lumbar scoliosis and trunk shift were statistically significant (*P* < 0.05).

**Table 1:** Comparison of preoperative, postoperative and final follow-up radiographic parameters

	Preoperative	Postoperative	Final follow-up	*P*
Trunk shift (mm)	34.7 ± 12.4	10.5 ± 7.2	8.4 ± 5.6	< 0.001
Thoracic scoliosis (°)	22.7 ± 10.6	6.2 ± 4.3	4.1 ± 4.3	< 0.001
Lumbar scoliosis (°)	18.1 ± 7.4	6.7 ± 5.2	5.3 ± 3.9	< 0.001
SVA (mm)	17.3 ± 12.5	15.7 ± 9.6	14.9 ± 10.3	0.614
TK (°)	27.4 ± 12.6	26.2 ± 11.9	26.5 ± 8.9	0.519
TLK (°)	7.2 ± 8.4	4.6 ± 7.3	4.8 ± 6.9	0.735
LL (°)	39.6 ± 13.7	39.2 ± 11.6	39.0 ± 10.8	0.682

There was no significant differences as to sagittal radiographic parameters (*P* > 0.05) (Table 1).

## Discussion

Lumbar spine was the most frequently affected region, followed by thoracic spine. Saffudin reviewed 78 English reports on spinal OO and osteoblastoma, and found 163 OOs at lumbar spine and 80 at thoracic spine [5]. We got similar results, and the ratio of lumbar spine to thoracic spine approximated 2:1. Most OO presented as isolated lesion below T8, and only one case of OO occurred at T6 in our cohort. OO distributed evenly at thoracic region, while been more likely seen at L3 and L4 for lumbar region. However, no matter where they occur, their curve patterns are similar. It demonstrated that pain-evoked muscle spasm had an effect on the whole spine no matter where it generates. Most OOs occur in the posterior element of the spine, including lamina, facet and pedicle. OOs occurring at the lamina tend to be unilateral and surround the facet. Similarly, no matter where OOs occur, curve pattern of scoliosis is similar.

**Fig. 1:**
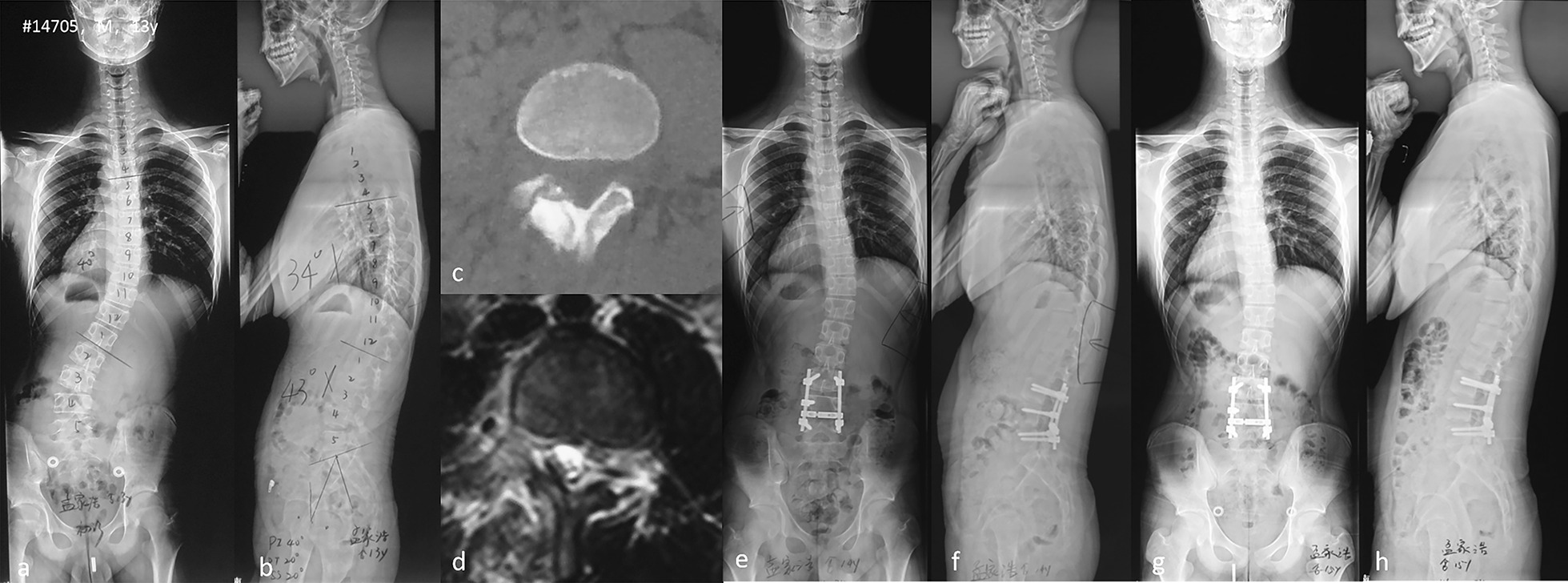
A 13-years-old male patient complained of back pain. X-rays demonstrated a 40° right thoracic scoliosis and a 28° left lumbar scoliosis (**a**) and normal sagittal alignment (**b**); CT demonstrated the nidus was located at right lamina (**c**), and MR showed edema around nidus (**d**); Thoracic scoliosis improved to 20°, and lumbar scoliosis improved to 8° at 3-month after surgery (**e**). Sagittal alignment was unchanged (**f**). Two-year follow-up X-rays showed that thoracic scoliosis was reduced to 16°, and lumbar scoliosis to 4° (**g**). No change of sagittal alignment was observed (**h**)

Scoliosis secondary to OO was always misdiagnosed as other spinal diseases such as idiopathic scoliosis and scoliosis associated with lumbar disc herniation [11]. Scoliosis secondary to OO and scoliosis associated with lumbar scoliosis were both pain-related. The pain of OO is localized and may be aggravated with motion. It is more severe at night and relieved by non-steroidal anti-inflammatory drugs (NSAIDs). Night pain was the characteristic manifestation of scoliosis secondary to OO, which played a great role in the diagnosis [2]. The etiopathogenesis of scoliosis associated with lumbar disc herniation was different. Nerve irritation and decrease of the weight-bearing capacity of healthy lower limbs were thought to contribute to scoliosis associated with disc herniation [12]. Therefore, the majority of the pain improved due to lying in bed, and there was no nocturnal pain. Another important differential diagnosis point was the relationship between location of pain and direction of curve. Pain of OO was always located at the concave side of scoliosis, while for lumbar disc herniation, pain could be located at both sides of scoliosis [13]. Sagittal alignment could also help differentiate scoliosis secondary to OO from scoliosis associated with lumbar disc herniation. Patients with scoliosis associated with lumbar disc herniation frequently display decreased lumbar lordosis and thoracic kyphosis, which are believed a pain-relief posture [14]. However, for scoliosis secondary to OO, we did not note this phenomenon. Patients presented similar sagittal alignment to normal population, and there were no significant difference of sagittal parameters before and after surgery (Fig. 1).

Nonsteroid anti-inflammatory drugs (NSAIDs) were recommended for pain control. However, it was not effective for scoliosis. Curettage was thought to be the standard surgical treatment for OO, which could obtain radical resection of nidus, thus halting the muscle spasm [15]. In our cohort, more than 70% patients got significant correction of scoliosis within 3 months. At last follow-up, all the patients obtained satisfactory outcomes with regards to both pain relief and scoliosis correction. The use of internal fixation mainly depends on the stability of the spine after curettage. In our cohort, only one patient received curettage without fixation, as lesion was located at midline of laminae without involving facet. The majority of the cases of our study had lesions at facet or pedicle, and curettage would inevitably damage the facet generating iatrogenic instability of the spine. Additional posterior fixation could permit long-term stability of the spine, especially for adolescents with great growth potential. One case had a lesion at vertebral body, and we performed curettage and fixation by anterior approach. Minimally invasive methods, such as CT-guided thermocoagulation and percutaneous radiofrequency ablation have also been adopted for the treatment of OO [16]. However, its effect on scoliosis remains to be observed. As a benign spinal tumor, the recurrence of OO is very low. In this study, no tumor recurrence was found at the last follow-up. Complete resection of the nidus can effectively avoid tumor recurrence.

In conclusion, patients with spinal OO had a significantly high incidence of scoliosis. Patients could get rapid relief of pain and scoliosis with low occurrence. Night pain, pain at the concave side of curve, normal sagittal alignment could help differentiate it from scoliosis associated with lumbar disc herniation.

## Data Availability

All datasets generated or analyzed during this study are available from the corresponding author on reasonable request.
